# Shining light on location-biased cAMP signaling

**DOI:** 10.1016/j.jbc.2021.101118

**Published:** 2021-08-23

**Authors:** Jean-Pierre Vilardaga, Ieva Sutkeviciute, Karina A. Peña

**Affiliations:** Laboratory for GPCR Biology, Department of Pharmacology and Chemical Biology, University of Pittsburgh School of Medicine, Pittsburgh Pennsylvania, USA

**Keywords:** adenylate cyclases, cAMP, endosomal signaling, G protein-coupled receptor, hormones, location bias, phosphoproteome, AC, adenylate cyclase, bPAC, bacteria-derived photoactivable adenylyl cyclase, GPCRs, G protein–coupled receptors

## Abstract

cAMP is the indispensable second messenger regulating cell metabolism and function in response to extracellular hormones and neurotransmitters. cAMP is produced *via* the activation of G protein–coupled receptors located at both the cell surface and inside the cell. Recently, Tsvetanova *et al.* explored cAMP generation in distinct locations and the impact on respective cell functions. Using a phospho-proteomic analysis, they provide insight into the unique role of localized cAMP production in cellular phospho-responses.

Similar to other extracellular stimuli, hormones and neurotransmitters transmit signals into cells by binding to different G protein–coupled receptors (GPCRs), which constitute the largest receptor family in the body. Peptidic and nonpeptidic hormones such as vasopressin and epinephrine, respectively, activate distinct GPCRs to induce the synthesis of cAMP, the second messenger molecule that activates PKA, which in turn regulates numerous metabolic functions (*e.g.*, glycogen metabolism, water homeostasis). The established paradigm of GPCR signaling *via* cAMP postulates that ligand binding to its cognate receptor induces conformational changes to the latter, enabling coupling and activation of the heterotrimeric G_s_ protein, the guanine nucleotide-binding protein that stimulates activity of transmembrane adenylate cyclase (AC) localized at the plasma membrane. Activated AC converts ATP into cAMP, a reaction that is rapidly terminated by the GTPase activity of G_s_ and by a cascade of reactions and interactions that desensitize the ligand-activated receptor, including (1) receptor phosphorylation, (2) interaction of the phosphorylated receptor with β-arrestins, and (3) internalization of the receptor–arrestin complex into early endosomes. Once in endosomes, the agonist dissociates from the receptor and the receptor is either degraded in lysosomes (downregulation) or dephosphorylated and recycled back to the plasma membrane for a new cycle of activation and signaling (resensitization) (reviewed in (1)).

Following initial studies on the parathyroid hormone and thyroid-stimulating hormone receptors back in 2009, another mode of cAMP signaling has been identified for several other GPCRs (reviewed in (1)). In this recently recognized model, receptors continue to signal through G_s_ after internalization into early endosomes to induce a second wave of cAMP production that not only causes unique pharmacological responses (2, 3) but also likely has physiological relevance for human health and disease (4, 5) (Fig. 1). Understanding molecular and cellular mechanisms of endosomal cAMP signaling thus has potential medical significance for the development of new therapies to treat bone and mineral diseases caused by hypocalcemia, a condition of low Ca^2+^ level in the blood (reviewed in (1, 3)). While essential mechanisms governing endosomal GPCR signaling *via* cAMP are emerging (6, 7), many key questions remain to be solved. One of these—how location bias in cAMP signaling can trigger unique cellular functions—has been recently addressed by Tsvetanova *et al.* (8).

**Figure 1:**
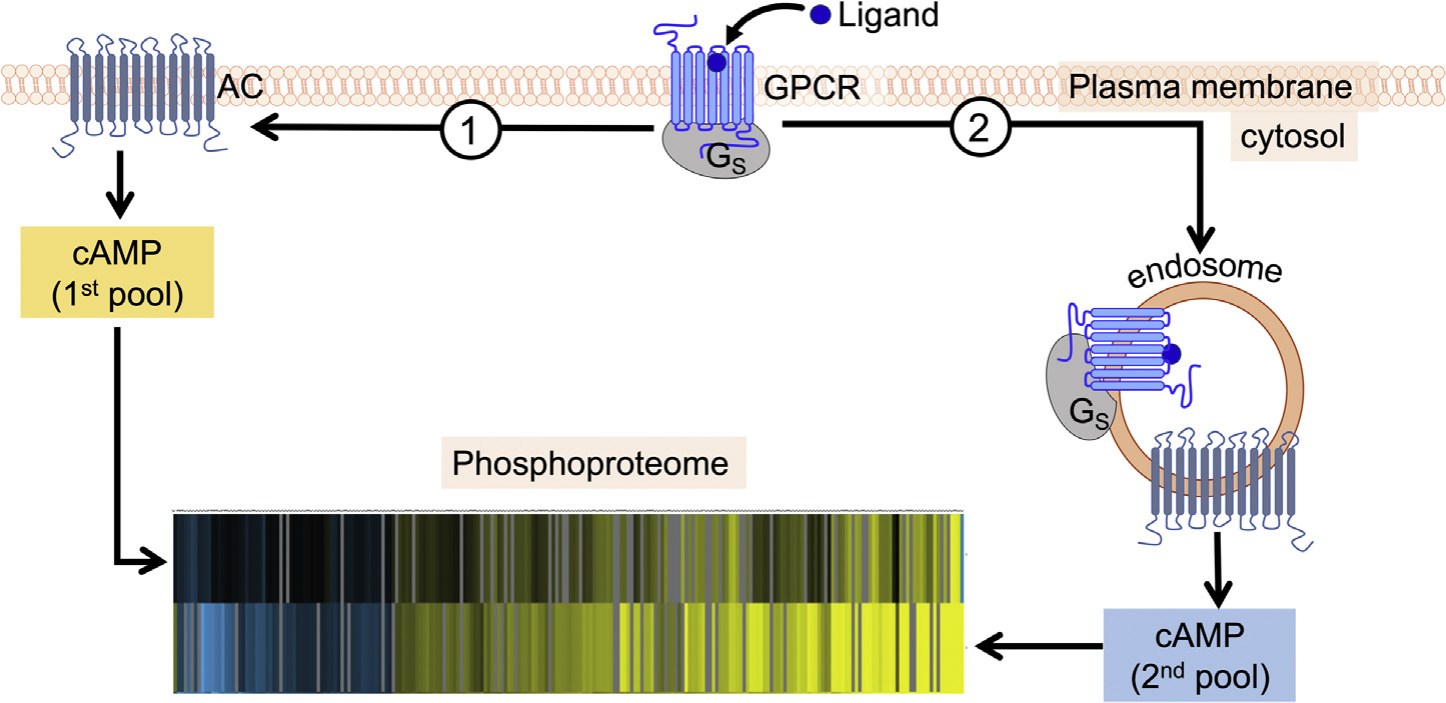
**Alternate modes of GPCR signaling *via* cAMP and the phosphoproteome.** The first pool of cAMP production takes place at the cell membrane after activation of G_s_ by the agonist-bound receptors (step 1). This cAMP response is usually short-lived because of the action of phosphodiesterases and rapid receptor desensitization, followed by receptor endocytosis (step 2). Eventually, internalized receptors induce a second pool of cAMP generation that originates from ligand–GPCR complexes in endosomes. Each location-derived cAMP pool triggers a unique phospho-proteomic response inside the cell. The phosphoproteome heat map is from Tsvetanova *et al.* (8). AC, adenylate cyclase; GPCR, G protein–coupled receptor.

One technical challenge when studying GPCR signaling is the difficulty in detecting compartmentalized cAMP production because of the dynamic membrane trafficking in live cells. To circumvent this issue, Tsvetanova *et al.* (8) used a previously described optogenetic approach in which bacteria-derived photoactivable adenylyl cyclase (bPAC) is targeted to distinct subcellular compartments, such as early endosomes, the cytosol, and the plasma membrane, to induce location-selective cAMP production after photo-stimulation by blue light. Because PKA is directly activated by cAMP, resulting in changes in the phosphorylation status of many downstream targets, the authors asked whether location bias of cAMP generation results in distinct or similar cellular phospho-proteomes. To this end, the authors applied a SILAC (Stable Isotope Labeling by Amino acids in Cell culture)-based proteomics approach to study the cellular phospho-proteome in human embryonic kidney 293 cells. The experimental principle is based on two populations of cells that are cultured side by side in media containing either “light” (^12^C,^14^N) or “heavy” (^13^C,^15^N) isotopic forms of arginine and lysine. “Light” cells, not photo-stimulated, serve as reference control. After photo-stimulation of “heavy” cells, lysates of an equal number of “light” and “heavy” cells are mixed, and phospho-peptides are then enriched by immobilized metal affinity chromatography and subjected to online LC/MS/MS analysis. Because ^13^C and ^15^N do not cause significant isotope effects, “light-” and “heavy”-labeled peptides are chemically identical but different in mass. This mass shift is detected by MS such that the ratio of peak intensities in the mass spectrum reflects the relative abundance of each phospho-peptide. This system proved to be reliable, given that one-third of all known GPCR/cAMP phosphorylation targets were found to be phosphorylated in response to cytosolic cAMP generated by cytosol-targeted bPAC. In addition, many of these targets contained the PKA target motif R-R/K-X-p(S/T), suggesting that all novel sites identified are likely to be linked to cAMP signaling.

The first insight of the study stems from the Gene Ontology and network analyses, unveiling that upon cytosolic cAMP production, a group of RNA-processing proteins become phosphorylated, a Gene Ontology category that has not been previously associated with cAMP signaling. This exciting finding will likely prompt more research toward better understanding of post-transcriptional regulation of GPCR signaling–mediated gene expression. The second insight is the observation that changes in protein phosphorylation are significantly higher when cAMP is generated from endosomes rather than the plasma membrane, despite very similar total amounts of cAMP generated at both locations (Fig. 1). However, the analysis of downregulated rather than upregulated phosphopeptides revealed protein phosphatase 2A to be a highly probable target of endosomal cAMP signaling, as its phosphorylation (and activation) at PKA target residue S573 occurred only in response to endosomal cAMP production. While mimicking the duration of cAMP generated by GPCRs *via* photo-stimulation of cAMP with the bPAC tool is challenging, the authors verified that bPAC phospho-proteomic data are consistent with those reporting the effect of receptor internalization upon phosphorylation of PKA substrates for two distinct GPCRs: the vasopressin type 2 receptor, known to evoke long-lasting endosomal cAMP production in recombinant and native cells (9), and the β_2_-adrenergic receptor, which triggers short-lived endosomal cAMP (10). In both cases, PKA-dependent phosphorylation is reduced when the receptor is retained at the plasma membrane. This result supports a previous study comparing the actions of location-biased vasopressin type 2 receptor signaling on phosphorylation of target proteins (9).

This study provides a valuable glimpse into possible cellular processes specifically regulated by endosomal cAMP. However, much remains to be learned. The next steps of this research will likely be (1) determining the mechanism by which location bias generation of cAMP causes distinct phospho-proteomic responses and (2) linking these responses to the regulation of physiological processes. The first will require studies involving, for example, the role of phosphodiesterases in the control of cAMP diffusion in cells. The second might be addressed, at least in part, by cell proteomics profiling of diverse tissues from small animals treated with biased GPCR ligands stimulating cAMP from either the cell surface or endosomes. From the work of Tsvetanova *et al.*, one overarching conclusion can be drawn. As location bias in cAMP signaling contributes to a primary cell function by regulating the phospho-proteomes, it shines the way to a better understanding of endosomal GPCR function *via* G proteins.

## Conflict of interest

The authors declare that they have no conflicts of interest with the contents of this article.
